# Multiplex polymerase chain reaction for pathogen detection in donor/recipient corneal transplant tissue and donor storage solution

**DOI:** 10.1038/s41598-017-06344-3

**Published:** 2017-07-20

**Authors:** Takehiro Hariya, Kazuichi Maruyama, Sunao Sugita, Masayo Takahashi, Shunji Yokokura, Kota Sato, Yasuhiro Tomaru, Norio Shimizu, Toru Nakazawa

**Affiliations:** 1 Department of Ophthalmology and Visual Science, Tohoku University Graduate School of Medicine, Sendai, Miyagi Japan; 2 Department of Innovative Visual Science, Osaka University Medical School, Suita, Osaka Japan; 3 Laboratory for Retinal Regeneration, RIKEN Center for Developmental Biology, Kobe, Hyogo Japan; 4 Department of Virology, Medical Research Institute, Tokyo Medical and Dental University, Tokyo, Japan

## Abstract

Corneal transplantation is a safe, reliable method of restoring visual acuity in patients with corneal disorders. Although it has a very high success rate, rejection can still occur, especially if the site is infected. Therefore, seeking to find better ways to manage infection risk, this study investigated a new technique, based on multiplex polymerase chain reaction (mPCR), to identify pathogens, including viruses, bacteria, and fungi, in corneal transplantation recipient sites, donor corneas and the donor cornea storage solution. The subjects comprised 50 patients who underwent corneal transplantation at Tohoku University Hospital between July 2014 and April 2015. We obtained extracted (recipient) cornea samples in 37 cases, donor cornea samples in 50 cases, and corneal storage solution samples in 50 cases (18 of these 50 samples contained DNA). *Herpes simplex virus type 1* DNA was detected in four recipient corneas, *Parvovirus B19* DNA was detected in two recipient corneas, *Human herpes virus type 6* was detected in two donor corneas, and *Aspergillus* DNA was detected in one corneal storage solution sample. Thus, mPCR successfully identified pathogenic DNA in corneal tissues and storage solution, suggesting that evaluation with mPCR may improve the ability to predict the risk of infection after corneal transplantation.

## Introduction

Corneal opacity is a common cause of blindness with many causes. Notably, corneal opacity can occur as a complication of corneal transplantation when the transplant site becomes infected. Recovery from such infection is extremely slow due to the low number of corneal blood vessels and corneal immune privilege^1^. Nevertheless, delayed treatment can allow new damage to the corneal layers, affecting corneal transparency. Effective treatment thus requires quick and accurate identification of the primary pathogen causing the infection. Therefore, the current study sought to establish new techniques for pathogen screening in a variety of tissue and material samples obtained during the perioperative period of corneal transplantation.

Corneal transplantation is a common ophthalmological procedure that is usually performed in response to reduced corneal transparency and has a high success rate^2^. Although the cornea is an immune privileged site, immunosuppressive drugs are often used to ensure that transplant rejection does not occur after corneal transplantation. Normally, in cases without new vessel formation, these drugs allow graft survival to reach 90%^2–4^. However, immunosuppressive drugs increase the risk of infection. Moreover, multiple infectious agents and vectors for such infection have been identified. Transmission of the *herpes simplex virus* (HSV) type 1 has been observed from the donor cornea to the recipient^5^. Furthermore, pre-existing HSV-1 infection in the recipient has been shown to increase the risk of rejection^6^. Finally, fungal infection in the donor storage solution has been reported to affect graft survival after transplantation^7^. These risks make it essential to be aware of infectious symptoms in the perioperative period.

Diagnosing corneal infection has long relied on microbial culturing and clinical examination^8^, techniques that remain gold standards. However, microbial culturing to detect corneal infections is limited by the extremely small volume of corneal samples. This prompted us to investigate polymerase chain reaction (PCR) analysis as an alternative, because it requires only a small sample volume and is already commonly used for diagnosis in other settings. Previous reports have shown that PCR can identify pathogenic agents in ocular tissue, including the aqueous humor and vitreous, and that it is clinically useful for the diagnosis of severe infections that cannot otherwise be identified^9^. An additional, important advantage of PCR is that it allows the use of multiplex techniques (mPCR) that can simultaneously test for multiple types of pathogen, including viruses, bacteria, and fungi, in a single sample.

This study set out to determine whether mPCR analysis could identify pathogens in corneal tissues and storage solution. mPCR can comprehensively, simultaneously test for many types of pathogen DNA, even in very small samples. To the best of our knowledge, this is the first report on the ability of mPCR analysis to reveal the presence of pathogen DNA in samples taken from donor corneas, corneal recipients and the donor storage solution during the perioperative period.

## Results

### Primary corneal disease in the patients

Table 1 shows primary corneal diseases and procedure types in the 50 patients included in this study. There were 13 cases of corneal opacity caused by interstitial keratitis, including 7 bilateral cases with suspected tuberculosis or measles and 6 unilateral cases with suspected herpes virus infection. There were 8 cases in which a previous graft had failed. The original disease in these cases included 2 cases of Fuch’s corneal dystrophy, 1 case of bullous keratopathy (BK) after trauma in child birth, 1 case of BK after radial keratotomy, 1 case of Reis-Buckler’s dystrophy and 3 cases of unknown origin. There were 3 patients with limbal stem cell deficiency, all of whom had previously undergone ocular surface surgery, including limbal transplantation (LT) and amniotic membrane transplantation. There were 2 cases of corneal perforation, both due to autoimmune disease. The average postoperative observation period was 11.5 ± 3.4 (6–18) months. The graft survival rates were 98% (*n* = 50), 94% (*n* = 50), 94% (*n* = 50), and 88% (*n* = 40), respectively, at 1, 3, 6, and 12 months postoperatively.

**Table 1:** Primary disease in patients undergoing corneal transplantation.

Clinical diagnosis	Primary disease	Number	Graft Fail
Interstitial keratitis	Bilateral (suspected tuberculosis or measles infection)	7	1
	Unilateral (suspected herpes infection)	6	0
Bullous keratopathy	Intraocular surgery	6	0
	Congenital anomaly	2	0
	Endotheliatis	2	0
	Laser iridectomy	1	0
	Trauma	1	1
	Trauma in child of birth	1	0
Re-operated eye	Graft failure	8	2
Dystrophy	Fuch’s	4	1
	Lattice	1	0
	Gelatinous drop-like	1	0
Palisades of Vogt deficiency	Stevens-Johnson syndrome	1	0
	Alkaline burn	1	0
	Idiopathic	1	0
Dermoid	—	2	0
Perforation	Autoimmunization	2	1
Keratoconus	—	2	0
Pseudo pterygium	Idiopathic	1	0
Total	—	50	6

### Investigation of pathogenic DNA in recipient and donor corneal tissue and storage solution

Sample types in this study included excised corneal tissue from the transplant recipients, material trimmed tissue from the donor corneas before transplantation, and the donor cornea storage solution. It was possible to extract DNA from all 50 donor cornea samples. However, it was possible to extract DNA from only 37 of the 50 recipient cornea samples, because 13 patients underwent Descemet’s stripping automated endothelial keratoplasty (DSAEK) or LT, procedures that do not allow for the collection of sufficient corneal tissue. Furthermore, it was possible to extract DNA from 18 of the 50 samples of donor cornea storage solution (Optisol; Chiron Ophthalmics, Irvine, USA), which is normally considered a sterile medium. Table 2 shows detailed PCR data for the samples from which DNA was extracted. Six of the 37 recipient samples (16%) were positive for HSV-1 or *human parvovirus B19*(parvo B19). Two of the 50 donor samples (4%) were positive for *human herpes virus* (HHV)-6 viral DNA. One sample of storage solution was positive for *Aspergillus* (6%).

**Table 2:** Infectious antigens in cornea recipients, donor corneas, and donor cornea storage solution.

	Age (recipient)	Gender	Microbe	Clinical diagnosis	Operative method	Sample Volume (µL)	OD (ng/µL)	Extracted Volume (µL)	Number of DNA copy (copies/ug or copies/ml)
Recipient									
A	69	M	HSV-1	Interstitial keratitis	DALK	200	12.2	100	3.9*10E2
B	84	M		BK (graft failure)	PK	200	9.3	100	9.6*10E2
C	76	M		Interstitial keratitis	DALK	200	42.2	100	2.7*10E1
D	64	M		Interstitial keratitis	PK	200	7	100	4.1*10E1
E	69	F	Parvo B19	Interstitial keratitis	DALK	200	14.3	100	5.7*10E2
F	75	F		Corneal perfoation	LK	200	23.4	100	4.8*10E2
Donor									
G	74	F	HHV-6	BK (LI)	DSAEK	200	17.9	100	1.2*10E2
H	80	F		PBK	PK	200	12.7	100	3.8*10E5
Storage solution									
I	55	M	Aspergillus 18S	BK (graft failure)	PK	400	1.4	60	8.6*10E5

BK = bullous keratopathy; DALK = deep anterior lamellar keratoplasty; DSAEK = Descemet’s stripping automated endothelial keratoplasty, HHV-6 = *human herpes virus type 6*; HSV-1 = *human simplex virus type 1*; LI = laser iridectomy; LK = lamellar keratoplasty; Parvo B19 = *human parvovirus B19*, PBK = pseudophakic bullous keratopathy; PK = penetrating keratoplasty.

**Table 3:** Conditions of the PCR method.

	Primer	Light Cycler	Taq	Annealing Temperature (°C)	Number of Cycles
Qualitative					
	Strip	2	Accu Prim Taq DNA Polymerase System	58	40
Quantitative					
	HHV1, 2	480II	Mix	60	45
	HHV 3–8				50
	Parvo B19	2			
	JCV, BKV, HBV				
	Bacteria 16S	480II	Ampli taq Gold		45
	Candida 18S	2	Mix		50
	Aspergirus 18S				

Mix = the monoclonal antibody for hot-start PCR anti-Taq high and Dream Taq DNA polymerase. BKV = *BK virus*; HBV = *hepatitis B virus*; HHV-1–8 = *human herpes virus type 1*–*8*; JCV = *JC virus*; Parvo B19 = *human parvovirus B19*.

**Figure 1:**
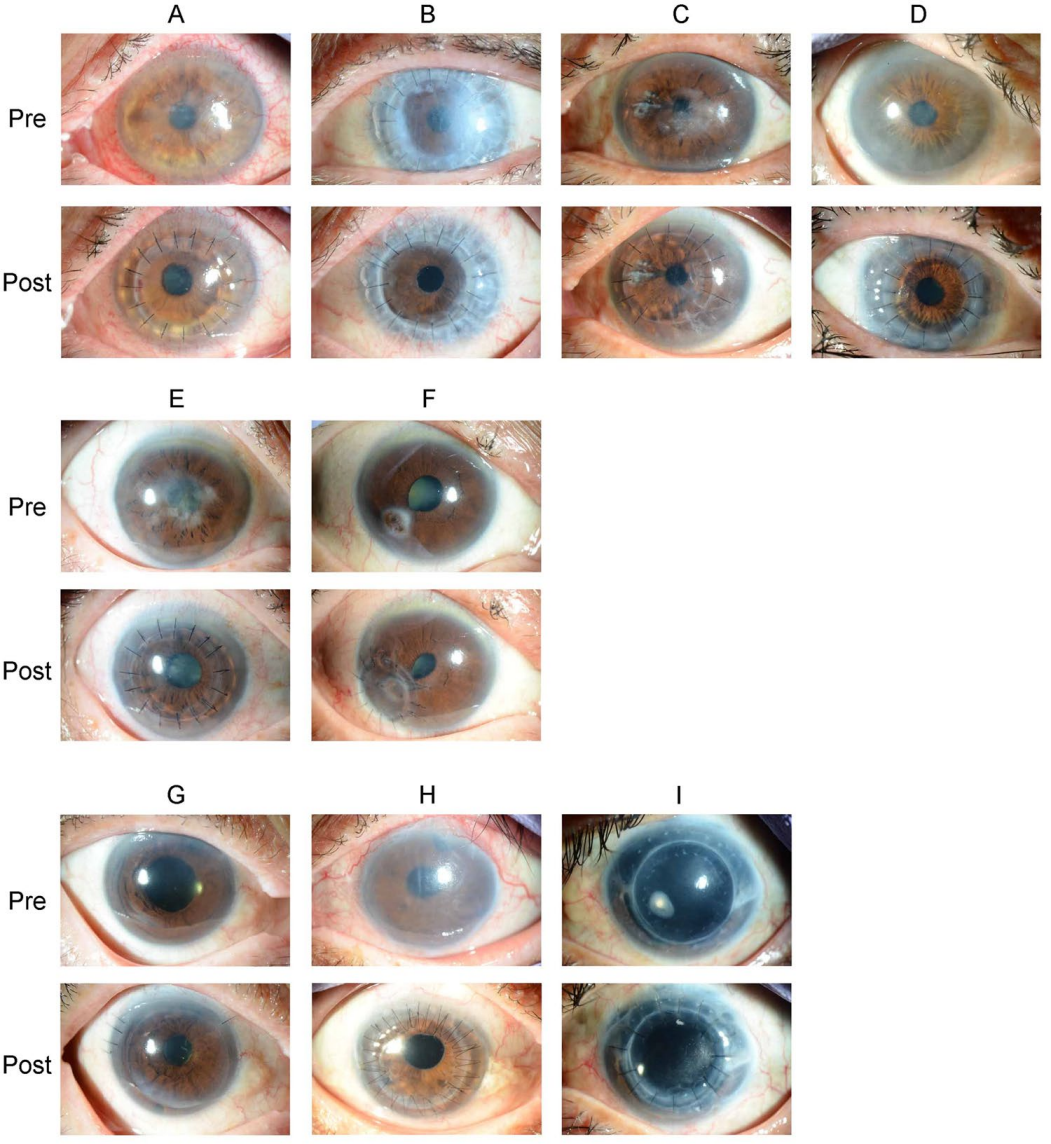
Details of positive PCR results in cornea recipient and donor samples. Images of the anterior segment in patients with positive PCR results. The upper images were obtained before surgery (pre) and the lower images were obtained after surgery (post). HSV-1 was detected in the extracted tissue of the herpetic keratitis patient (**A**–**D**). Parvo B19 was detected in the extracted tissue of the patient with bilateral corneal opacity after interstitial keratitis (**E**,**F**). HHV-6 was detected in the donor tissue in the patient treated for bullous keratopathy after both laser iridectomy and intraocular surgery (**G**,**H**). *Aspergillus* was detected in the storage solution of the donor cornea used in the patient treated for bullous keratopathy after graft failure (**I**). HHV-6 = *human herpes virus type 6;* HSV-1 = *human simplex virus type 1;* Parvo B19 = *human parvovirus B19*; PCR = polymerase chain reaction.

The recipient cornea samples that were positive for HSV-1 were obtained from three patients with interstitial keratitis and one patient with graft failure. These patients all received antiviral treatment with acyclovir topical ointment 3 times per day, and had good postoperative clinical courses (Fig. 1A–D). The samples that were positive for parvo B19 were obtained from one patient with interstitial keratitis and one patient with corneal perforation. Both patients had good postoperative clinical courses without the need for any antiviral medications (Fig. 1E,F).

Two donor corneal samples were positive for HHV-6 DNA. We investigated the background of these donors but found no record of their HHV-6 status. The recipients of these donor corneas included one patient with BK (caused by laser iridectomy) and one patient with pseudophakic BK (PBK). The BK patient underwent DSAEK, while the PBK patient underwent penetrating keratoplasty (PK). Neither patient received any viral medication after the corneal transplantation. Despite the positive result for HHV-6, both patients had a good postoperative clinical course (Fig. 1G,H).

Among the 18 samples of storage solution that were positive for any kind of DNA (36%), only 1 was positive for pathogen DNA (*Aspergillus*). However, the donor cornea that had been held in this storage solution did not test positive for *Aspergillus*. After corneal transplantation, the patient showed no signs of infection, experienced any other adverse events, and received no antifungal treatment (Fig. 1I).

## Discussion

This study showed that mPCR analysis can reveal the presence and type of pathogen DNA in three vectors of infection after corneal transplantation: the recipient, the donor cornea, and the donor cornea storage solution. Generally, corneal transplantation has been reported to have a high success rate, and our study reflected this, achieving a success rate of 88% at 12 months with a variety of transplantation techniques^2^. Despite this success, mPCR showed that pathogen DNA was present in many of the tissues and materials used in these procedures. We consider that this result demonstrates the potential of mPCR analysis to improve the safety and success of corneal transplantation. Ideally, corneal tissue and other materials should be pathogen-free during transplantation. Indeed, most donor corneal samples in this study were negative for the DNA of common pathogens, such as herpes virus keratitis. However, postoperative infection is a serious, sight-threatening condition, and the technique described here promises to help identify patients needing preventive treatment or more careful postoperative observation and thereby reduce the risk of corneal transplantation failure.

The methodology of this study used mPCR to screen for the DNA of multiple pathogens simultaneously, including bacteria, fungi, and viruses, such as herpes. After mPCR revealed the presence of pathogen DNA, real-time PCR was used to measure the DNA load of the specific pathogen. This made it possible to obtain quantitative information on the level of pathogen DNA in the samples. The broad-range real-time PCR system used here can detect DNA from many types of pathogen, including bacterial 16S, and *Candida* or *Aspergillus* 18S rDNA^10^. Diagnostic methods based on this system promise to be useful in clinical situations in which it is unclear whether the nature of a corneal infection is bacterial or fungal. Moreover, real-time PCR plays a key role in preventing contamination from affecting diagnostic accuracy, since quantitative results can be used to confirm the presence and quantity of pathogen DNA after its presence has been qualitatively identified with mPCR^9^. Previous studies have shown that comprehensive PCR examination allows the highly accurate diagnosis of ocular infection, with sensitivity, specificity, positive predictive values, and negative predictive values all reported to be over 90%^9^.

The pathogen DNA identified in this study included HSV-1, parvo B19, HHV-6, and *Aspergillus*. We closely followed all patients with positive results, but we did not observe any variation from the normal, post-corneal transplantation clinical course. This asymptomatic course may have occurred for two reasons. First, the recipient samples that were infected with HSV-1 had a copy number of 2.7X10E–9.6X10E2, which is relatively low. Second, despite this low copy number, the HSV-1-positive patients were treated prophylactically with antiviral drugs, due to the presentation of a relevant clinical appearance.

The patients with positive results for parvo B19 DNA also had a good postoperative course, but the ophthalmic symptoms of parvo B19 are not yet well known, despite findings that the parvo B19 virus underlies fifth disease^11^. Thus, it is possible that parvo B19 infection had an unobserved impact on our patients. Continued careful observation of these patients is therefore necessary. Similarly, the patients with positive results for HHV-6 DNA showed no symptoms after transplantation and were not treated with any antiviral drugs. HHV-6 is known as the cause of idiopathic eruption^12^, and in the field of ophthalmology, it has been reported that HHV-6 can induce severe unilateral panuveitis when the immune system is compromised^13, 14^. Two donor corneal samples in this study were positive for HHV-6 DNA, both of which came from donors who were not immunocompromised and did not have any adverse ocular conditions. However, HHV-6 infection in the cornea has not been studied, and there are no reports to guide treatment. Moreover, during the follow-up period, we did not obtain conclusive evidence on whether viral replication of HHV-6 had occurred in the corneas. Therefore, continued evaluation of the clinical course of these patients is necessary.

One sample of the donor cornea storage solution was positive for *Aspergillus* DNA, but fortunately, the recipient of this cornea showed no postoperative infectious symptoms. Furthermore, the extracted (donor) cornea of this patient was negative for *Aspergillus* DNA. This patient continues to visit our outpatient clinic for the treatment of severe dry eye, which has allowed us to gather additional data for inclusion in this report. We hope to continue observation of this patient and perform further testing, if necessary.

This study was limited by its short observation period, which may explain why despite the positive results we obtained for pathogen DNA in the samples, the patients showed no symptoms of infection after transplantation. Therefore, we plan to continue observing the patients with positive DNA results. Additionally, this study may have been affected by false-positive and false-negative results caused by the exposed nature of the ocular surface, which can cause sample contamination. However, this is a general limitation of DNA testing of ocular tissue samples, and it is important to remember that contamination with non-disease-causing pathogens can always occur during preparation for PCR testing. Therefore, in general, PCR analysis should always be used in combination with multiple clinical examinations to most accurately diagnose corneal disease.

In conclusion, this study showed that PCR analysis can detect the DNA of pathogenic agents in corneal tissue extracted from transplant recipients and donors, as well as the donor cornea storage solution. Thus, the technique described here promises to improve the safety of corneal transplantation and increase its success rate. This study also made the interesting finding that viral DNA of unknown origin was present in cornea samples obtained during the transplantation perioperative period. This suggests that in the future, our technique might reveal new information on infectious diseases that are transmissible through corneal transplantation. Our technique also has the potential to improve quality management in various fields of medicine, such as regenerative medicine and the production of cultured cell sheets. Thus, the results of the present study indicate that mPCR analysis is a useful way of quickly identifying patients at risk of post-transplantation corneal infection, potentially improving the timeliness and accuracy of treatment for these patients.

## Methods

### Patients

This study recruited 50 patients who underwent corneal transplantation at Tohoku University Hospital between July 2014 and April 2015. Samples of the recipient corneal beds and donor corneas, as well as the donor cornea storage solution (Optisol), were collected from these patients. The average patient age at the time of surgery was 65 ± 18 (17–85) years. Twenty-five patients were male and 25 were female. The average donor age was 63 ± 13 (13–95) years old. We used 46 donor corneas imported from a foreign commercial eye bank (Sight Life, Seattle, USA) and 4 donor corneas obtained domestically. This study used several corneal transplantation methods, including PK, deep anterior lamellar keratoplasty (DALK), lamellar keratoplasty (LK), DSAEK, and LT.

After keratoplasty, surplus tissue from the donor corneas was collected for use in the PCR analysis. Extracted corneal tissue was collected for the PCR analysis in patients who underwent the following types of keratoplasty: PK, DALK and LK. PCR was also used to analyze 1.0-ml samples of the donor cornea storage solution. All samples were preserved in pre-sterilized microfuge tubes at 4 °C and transferred to a laboratory, located in Kobe, which is approximately 600 km distant from our facility. To ensure that the samples were free of contamination before the PCR analysis, separate laboratories performed DNA amplification and analysis of the amplified products, according to a method reported in a previous study^15^.

The research followed the tenets of the Declaration of Helsinki. The Institutional Ethics Committee of Tohoku University Hospital approved all study protocols. The clinical trial was registered (incorporating revisions to the study protocol) on February 25, 2016 (UMIN000004980 and 000021204). The purpose of this research and the experimental protocols (including both study participation and publication) were explained in detail to all patients, and their informed consent was obtained prior to participation in this study.

### PCR Analysis

**Figure 2:**
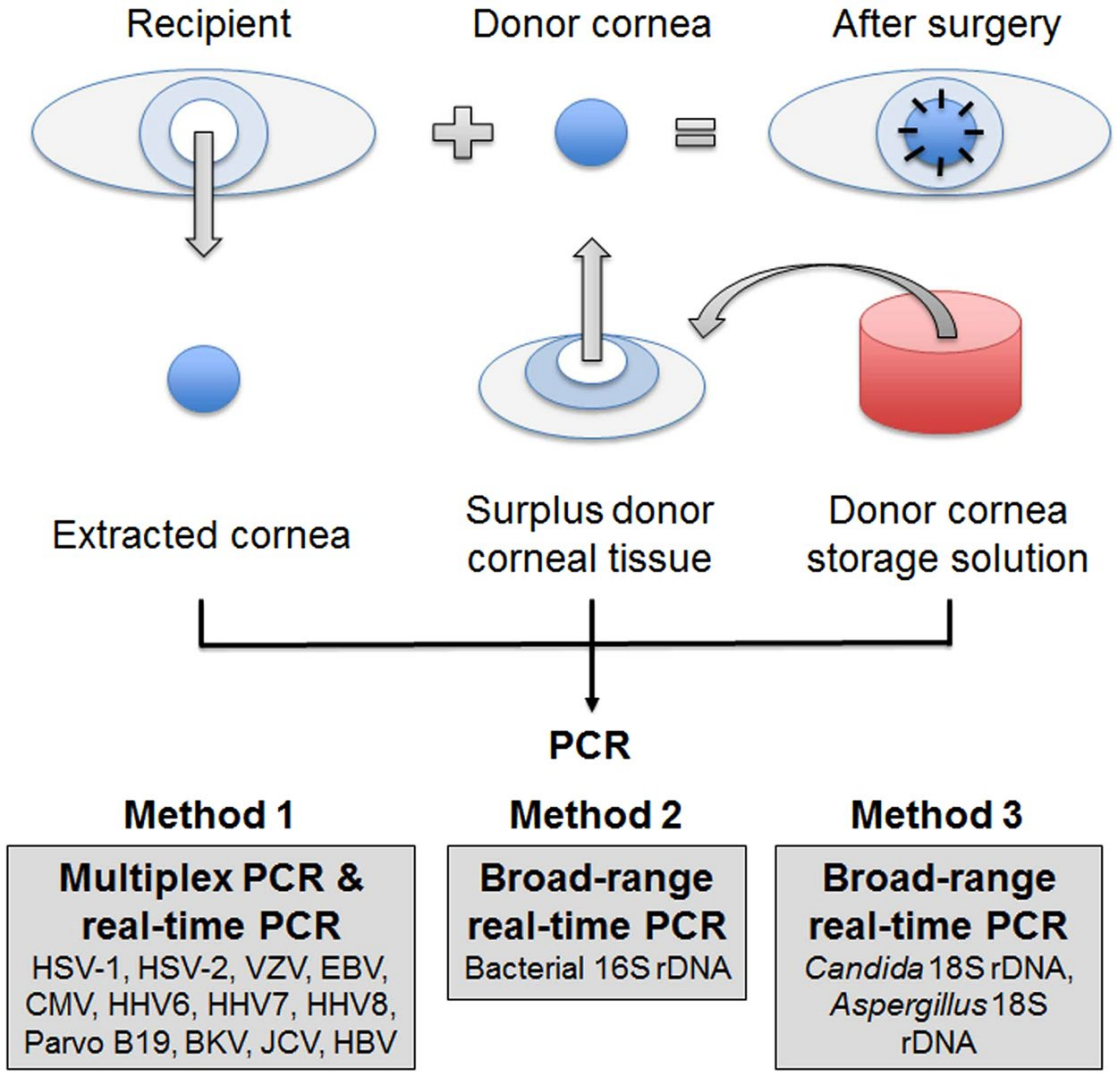
Diagram of method for comprehensive PCR analysis of pathogen DNA in extracted recipient corneal tissue, donor corneal tissue, and donor cornea storage solution. This method comprised the three following steps: (1) qualitative multiplex PCR and quantitative real-time PCR, (2) broad-range real time PCR, and (3) broad-range real time PCR. Method 1: DNA from the following pathogens was found in the extracted tissue, the donor tissue and storage solution: HSV-1, HSV-2, VZV, EBV, CMV, HHV-6, HHV-7, HHV-8, BKV, JCV and HBV. Method 2: broad-range real time PCR in the extracted tissue, donor tissue, and storage solution revealed the presence of bacterial 16s rDNA. Method 3: broad-range real time PCR in the donor cornea storage solution revealed the presence of *Candida* and *Aspergillus* 18s rDNA. BKV = *BK virus*; HBV = *hepatitis B virus*; CMV = *Cytomegalovirus*; EBV = *Epstein–Barr virus*; HHV-6-8 = *human herpes virus type 6-8*; HSV-1, 2 = *herpes simplex virus type-1*, *2*; JCV = *JC virus*; Parvo B19 = *human parvovirus B19;* PCR = polymerase chain reaction; VZV = *varicella-zoster virus*.

The sampling procedure and the PCR methodology are shown in Table 3﻿ and﻿ Fig. 2. DNA was extracted from the corneal samples with a DNA Mini kit (Qiagen, Valencia, USA) and from the liquid samples with a Virus Spin kit (Qiagen). The total volume of extracted DNA was 100 ng for each type of PCR. The analysis used Taqman-type probes, with GAPDH as an internal control. Genomic DNA for HHV, parvo B19, *BK virus* (BKV), *JC virus* (JCV), *hepatitis B virus* (HBV), bacteria, and fungi (*Candida* spp. & *Aspergillus* spp.) was measured with two independent PCR assays: the first used a combination of qualitative mPCR and quantitative real-time PCR, and the second used only broad-range real-time PCR (Fig. 2).

The first assay was used for the viral species: HSV-1 (HHV-1), HSV-2 (HHV-2), *varicella-zoster virus* (VZV; HHV-3), *Epstein–Barr virus* (EBV; HHV-4), *cytomegalovirus* (CMV, HHV-5), HHV-6, HHV-7, HHV-8, Parvo B19, BKV, JCV, and HBV. The first step comprised an mPCR assay using the Light Cycler 2.0 (Roche, Basel, Switzerland) and the Accu Prim Taq DNA Polymerase System (Thermo Fisher Scientific, Waltham, USA) to qualitatively measure genomic DNA. The PCR method and materials described here were tested on standard control pathogenic DNA that was obtained commercially (Nihon Techno Service, Ibaraki, Japan). If the mPCR results were positive, we then proceeded to the second step: real-time PCR with the Light Cycler 480 II (Roche) for HHV 1–2 and the Light Cycler 2.0 for all other viral species. Real-time PCR analysis of the viral species used the hot-start PCR method, including an anti-Taq monoclonal antibody (Toyobo, Osaka, Japan) and Dream Taq DNA polymerase (Thermo Fisher Scientific, Waltham, USA).

The second assay used broad-range real-time PCR to quantitatively measure the genomic DNA of bacteria 16S, *Candida* 18S, and *Aspergillus* 18S. The analysis of bacteria 16S used the Light Cycler 480 II and Amplitaq Gold (Thermo Fisher Scientific, Waltham, USA), a method we have previously described^15^. The analyses for *Candida* and *Aspergillus* used the Light Cycler 2.0 with the same antibody and Taq polymerase.

Primers and probes for HHV types 1–8 and the PCR conditions have been described previously^16, 17^, as have the primers and probes for the other viruses^18^ and the primers and probes for *Candida* 18S and *Aspergillus* 18S^10, 19–21^. Amplification of the human β-globulin gene served as an internal positive extraction and amplification control. Significant differences in copy number were defined as differences greater than 10 copies/μg or ml for *Candida* 18S and *Aspergillus* 18S; more than 50 copies/μg or ml for HHV-1-8, parvo B19, BKV, JCV, and HBV, and more than 100 copies/μg or ml for bacterial 16S^9^.
